# Optimizing operating room efficiency in robotic‐assisted total knee arthroplasty through manufacturing efficiency principles

**DOI:** 10.1002/jeo2.70283

**Published:** 2025-05-26

**Authors:** Akito Hiraoka, Bart Swinnen, Aline Vandeputte, Willem Franssen, Geert Leirs

**Affiliations:** ^1^ Department of Orthopaedics and Traumatology Noorderhart Hospital Pelt Belgium; ^2^ Department of Orthopaedics and Traumatology University Hospitals Leuven Leuven Belgium; ^3^ DEO.care Beringen Belgium

**Keywords:** data‐driven technology, operating room efficiency, process optimization, robotic‐assisted total knee arthroplasty, value‐based healthcare

## Abstract

**Purpose:**

Recent studies have explored the application of manufacturing efficiency principles as a framework for improving operating room (OR) throughput, quality of care and patient outcomes. However, these principles have not yet been validated with real‐world research data. In this study, we investigated whether applying these principles has an impact on the operational excellence and teams' consistency of robotic‐assisted total knee arthroplasty (RATKA) procedures.

**Methods:**

A total of 31 patients, divided over eight surgery days, were included in the study. The aim of the study was to answer the research question: ‘Does applying efficiency principles have an impact on the operational excellence of RATKA surgery?’ The efficiency of the procedures was monitored using the artificial intelligence (AI)‐enabled camera application and analyzed utilizing an AI‐backed process digital twin platform. Normally distributed continuous variables were compared using the independent *t* test. Equality of variances was assumed using the Levene's test for equality of variances. Non‐normally distributed continuous variables were analyzed using the Mann–Whitney *U* test.

**Results:**

After analyzing the baseline group, four procedural modifications were implemented according to the lean principles. There was a significant difference in preparation time (mean difference = 4.3, 95% confidence interval = 1.3–7.3, *p* = 0.007), showing the efficiency gain during preparation after efficiency implementation. The number of sets opened per case was reduced from ten to seven sets.

**Conclusions:**

Findings indicate that better OR preparation, consistent staff allocation and effective staff training can reduce surgical times, minimize waste and improve OR throughput. Addressing primary constraints, parallelizing flows and breaking down processes decreases surgical wait times, enhances patient flow and streamlines OR operations.

**Level of Evidence:**

Level III.

**Abbreviations**
AI: artificial intelligence
BMI: body mass index
CI: confidence interval
Deg.: degrees
IQR: interquartile range
LDFA: lateral distal femoral angle
MD: mean difference
MHKA: mechanical hip–knee–ankle angle
MPTA: medial proximal tibial angle
n.s.: not significant
OR: operating room
RATKA: robotic‐assisted total knee arthroplasty
SD: standard deviation
TKA: total knee arthroplasty

## INTRODUCTION

Operating rooms (ORs) are a critical financial component of hospitals. Surgical care represents about a third of all healthcare spending and surgical procedures themselves account for approximately 60% of the operating cost of a hospital [15, 17]. Therefore, optimizing and maximizing OR efficiency has been a discussion point for decades and are valuable strategies for lowering hospital expenses [2, 3, 9, 10, 22].

OR efficiency is typically driven by reducing non‐operating time and standardizing the OR processes. It not only has implications for reducing total cost of care, but also for improving patient experience, quality of care and ensuring a healthy and sustainable work environment [18, 20]. A holistic approach is essential, addressing every phase of the procedure—preparation, surgery, breakdown, and cleaning—rather than concentrating solely on the surgical phase.

Total knee arthroplasty (TKA) is among the most frequently performed procedures in orthopaedic surgery [1, 12, 13]. The advent of robotic‐assisted TKA (RATKA) could lead to improved functional outcomes due to the precise placement of components [6, 14]. However, the cost‐effectiveness of RATKA compared to manual TKA remains controversial, with current literature being limited and inconclusive [11]. Despite this, many hospitals are striving to enhance the value and reduce the costs associated with RATKA by improving both interoperative and intraoperative efficiency.

Sershon et al. [21] and DeCook et al. [5] published about manufacturing efficiency principles to revolutionize OR management, as a model for enhancing OR throughput, quality of care, and patient outcomes. The efficiency principles tackled in this study are ‘time transparency’, ‘the theory of constraints’, ‘parallel tasks’ and ‘work breakdown structure’.

‘Time transparency’ is understood as effective allocation and utilization of time resources, which is crucial in the setting of the OR. By providing a clear view of how time is expended across various tasks and processes, an organization can uncover critical insights into operational dynamics. The ‘theory of constraints’, on the other hand, explains how any team member has the potential to become a bottleneck that slows down the process [5]. It is thought that by having dedicated teams, specialized in specific procedures, we can reduce OR time and improve efficiency [8, 19]. Furthermore, within the OR, serial tasks often lead to inefficiencies; every sequential delay amplifies, extending wait times and reducing throughput. Implementing ‘parallel tasks’ in healthcare can markedly improve operational flow [5]. Additionally, breaking down the entire OR process, as the ‘work breakdown structure’ principle describes, helps identify which small actions affect OR flow and process efficiency. It allows staff to critically look at each part of their contribution to the flows and identify waste, redundancy and ways to improve each individual step that is required [21].

The principles above have not yet been validated in the literature with real‐world research data. This study explored the impact of applying efficiency principles on the operational excellence of surgery, specifically in RATKA procedures. Data‐driven digital twin technology was used to systematically collect data on operative time, staff activities and instrument usage.

## MATERIALS AND METHODS

### Cohort

A total of 31 patients, divided over eight surgery days, were included in the study from October 2023 until May 2024, all undergoing RATKA, performed by a single surgeon. Inclusion criteria were adult men and women with end‐stage osteoarthritis (Kellgren–Lawrence Grades III and IV). All participants provided informed consent, and the study was approved in advance by the institutional ethical board (CME Noorderhart).

### Study design

The aim of the study was to answer the research question: ‘Does applying efficiency principles have an impact on the operational excellence of RATKA surgery?’

Twenty‐five patients divided over seven OR days in October 2023 were assigned to the baseline measurements group, and six patients of one OR day in May 2024 were assigned to the post‐implementations group after a data‐driven optimization of the processes were performed, introducing efficiency implementation based on efficiency principles.

### Surgical technique

All patients underwent surgery via a medial subvastus approach utilizing the MAKO robotic‐arm system (Stryker) for RATKA. The Triathlon cruciate‐retaining implant was used in all cases. Both tibial and femoral cuts were executed using a functional alignment strategy. The decision to use cemented or uncemented implants was made intraoperatively by the senior surgeon, based on the patient's sex, age and bone quality. High‐viscosity cement with Gentamycin (Heraeus Palacos) was employed for cemented implants. Patellar resurfacing was performed in all cases. Closure of the capsule and subcutaneous tissue was achieved with interrupted absorbable sutures, and skin closure was completed using skin staples. No tourniquet was used for all patients. All procedures of one OR day were performed in the same OR room.

### Data collection

The efficiency of the RATKA procedures was monitored using the AI‐enabled camera application of an independent company (DEO.care). Seventy‐five RATKA‐specific timestamps were captured per case by an experienced Medical Engineer using the DEO.care AI‐backed process digital twin platform.

The total OR time in this study was calculated as the interval from the first activity happening in the OR until the end of the last activity. For the procedures under investigation, the first activity was the start of sterility, meaning opening of the sterility pack, since the opening of the sterility pack started before the patient entered the OR. The last activity happening was the patient leaving the OR.

**Figure 1:**
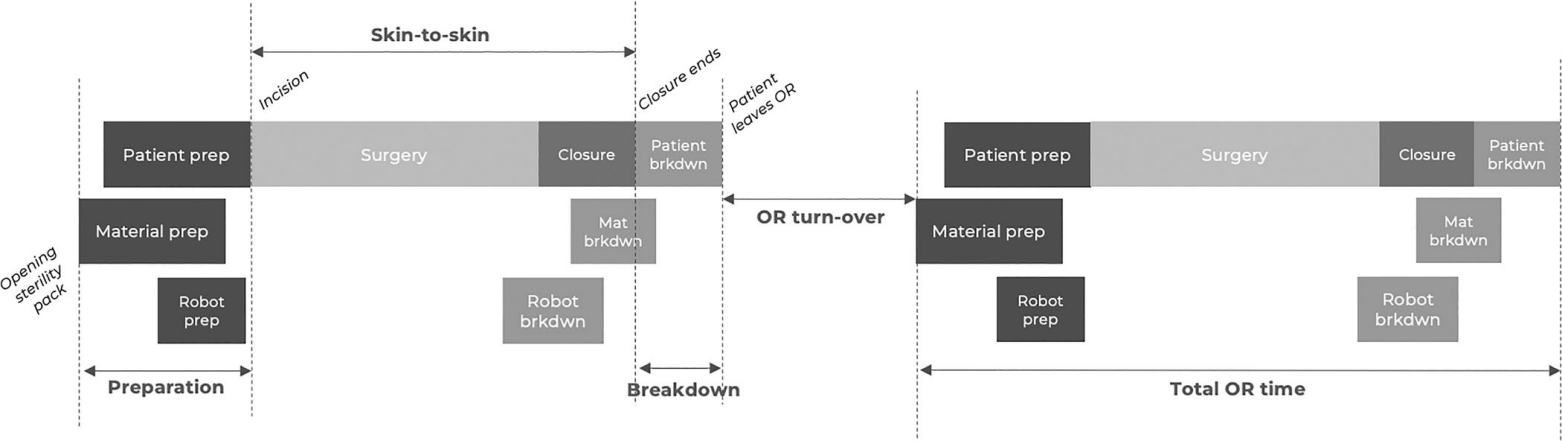
Definitions.

For each procedure, four major phases were differentiated through the timestamps (Figure 1):

1. Preparation: The interval from the first activity happening (opening of the sterility pack) until incision, covering the preparation of the patient, the instruments and the robot.
2. Skin‐to‐skin (surgery + closure): The interval from incision until the end of wound closure.
3. Breakdown: The interval from the end of wound closure until the last activity (the patient leaving the OR).
4. OR turn‐over: The interval from the last activity happening (the patient leaving the OR) until the first activity of the next procedure (next case's opening of the sterility pack).

Additionally, data on instrument usage and staffs OR presence, tasks and responsibilities were collected. OR presence of the staff was collected by annotating the stakeholder present in the digital twin tool. The tasks and responsibilities during the procedures were collected by linking staff to tasks. Data on instrument usage were collected by mounting the AI‐enabled camera application on top of the instrument table. Then, each used instrument was annotated using the AI‐backed process digital twin platform.

### OR team

The OR teams consisted of five people, including an experienced surgeon, an instrumenting nurse, a circulating nurse, an anaesthesiologist and a sales representative. The surgeon and the instrumenting nurse were sterile during the procedure. The instrumenting nurse and the sales representative performed the robot preparation. The sales representative performed the robot breakdown.

With the exception of the surgeon and the sales representative, the composition of the OR team varied from day to day. The OR team was assigned by the OR manager without input from the surgeon. The instrumenting nurse and circulating nurse alternated roles between procedures on the same day.

### Statistical analysis

Continuous variables, including time metric data, body mass index (BMI), age and coronal alignment parameters (mechanical hip–knee–ankle angle, lateral distal femoral angle and medial proximal tibial angle) were described using means, standard deviations (SDs), ranges, medians and interquartile ranges (IQRs). The other categorical variables were summarized as numbers and percentages. Initial analyses were conducted to evaluate baseline differences in cohort characteristics between the groups, ensuring no significant differences existed. Subsequently, analyses focused on comparing time metric data to assess changes in efficiency following the implementation of the data‐driven procedural changes.

The Shapiro–Wilk test was used to assess the normality of continuous variables. Normally distributed continuous variables were compared using the independent t‐test, with statistical significance set at a *p* value of <0.05. Equality of variances was assumed using the Levene's test for equality of variances. Non‐normally distributed continuous variables were analyzed using the Mann–Whitney *U* test, with statistical significance set at a *p* value of <0.05. Effect sizes for differences in continuous variables were reported as mean differences (MDs) along with their 95% confidence intervals (CIs). All statistical analyses were performed using SPSS (V29.2.0).

### Efficiency implementation

Efficiency was optimized using the DEO.care's best practice database and by focusing on the efficiency principles described by Sershon et al.^21^ and DeCook et al.^5^

## RESULTS

### Cohort characteristics

Cohort characteristics are described in Table 1. The mean age was 69.0 ± 6.8 y/o and 69.7 ± 7.2 y/o, respectively, for the baseline group and the post‐implementation group, while the BMI was 30.1 ± 7.7 kg/m^2^ and 29.8 ± 4.5 kg/m^2^ (*p* = not significant), respectively, for the baseline group and the post‐implementation group. There were no statistically significant differences in age, BMI or coronal alignment parameters between the baseline and post‐implementation groups.

**Table 1:** Cohort characteristics.

	Baseline				Post‐implementation				Effect size		
Characteristics	Mean ± SD, *n* (%)	Range	Median	IQR	Mean ± SD, *n* (%)	Range	Median	IQR	*p*	MD	95% CI
Age (years)	6.9 ± 6.8	58–80	69	63–73	69.7 ± 7.2	58–78	71.0	66.5–74.0	n.s.	0.3	−4.1 to 4.7
BMI (kg/m^2^)	30.1 ± 7.8	22–40	31	26–34	29.8 ± 4.5	24–35	29.0	27.3–33.8	n.s.	−0.7	−7.1 to 5.7
Sex											
Women	12 (48%)				3 (50%)						
Men	13 (52%)				3 (50%)						
Side											
Left	10 (40%)				5 (83%)						
Right	15 (60%)				1 (17%)						
Cementation											
Cemented	13 (52%)				3 (50%)						
Uncemented	12 (48%)				3 (50%)						
LDFA (deg.)	86.6 ± 2.5	80.4–90.5	86.8	85.0–88.5	87.2 ± 3.7	81.2–92.2	87.3	84.6–90.0	n.s.	−0.6	−3.1 to 1.9
MPTA (deg.)	86.9 ± 2.0	83.4–91.7	87.1	85.2–88.5	88.0 ± 2.1	84.9–90.5	87.9	86.3–90.2	n.s.	−1.1	−3.0 to 0.8
MHKA (deg.)	176.8 ± 4.0	167.5–185.0	177.0	175.3–178.8	177.7 ± 184.5	172.0–184.5	176.0	173.9–183.4	n.s.	−0.9	−4.8 to 3.0

Abbreviations: BMI, body mass index; CI, confidence interval; Deg., degrees; IQR, interquartile range; LDFA, lateral distal femoral angle; MD, mean difference; mHKA, mechanical hip–knee–ankle angle; MPTA, medial proximal tibial angle; n.s., not significant; SD, standard deviation.

### Change implementation

**Figure 2:**
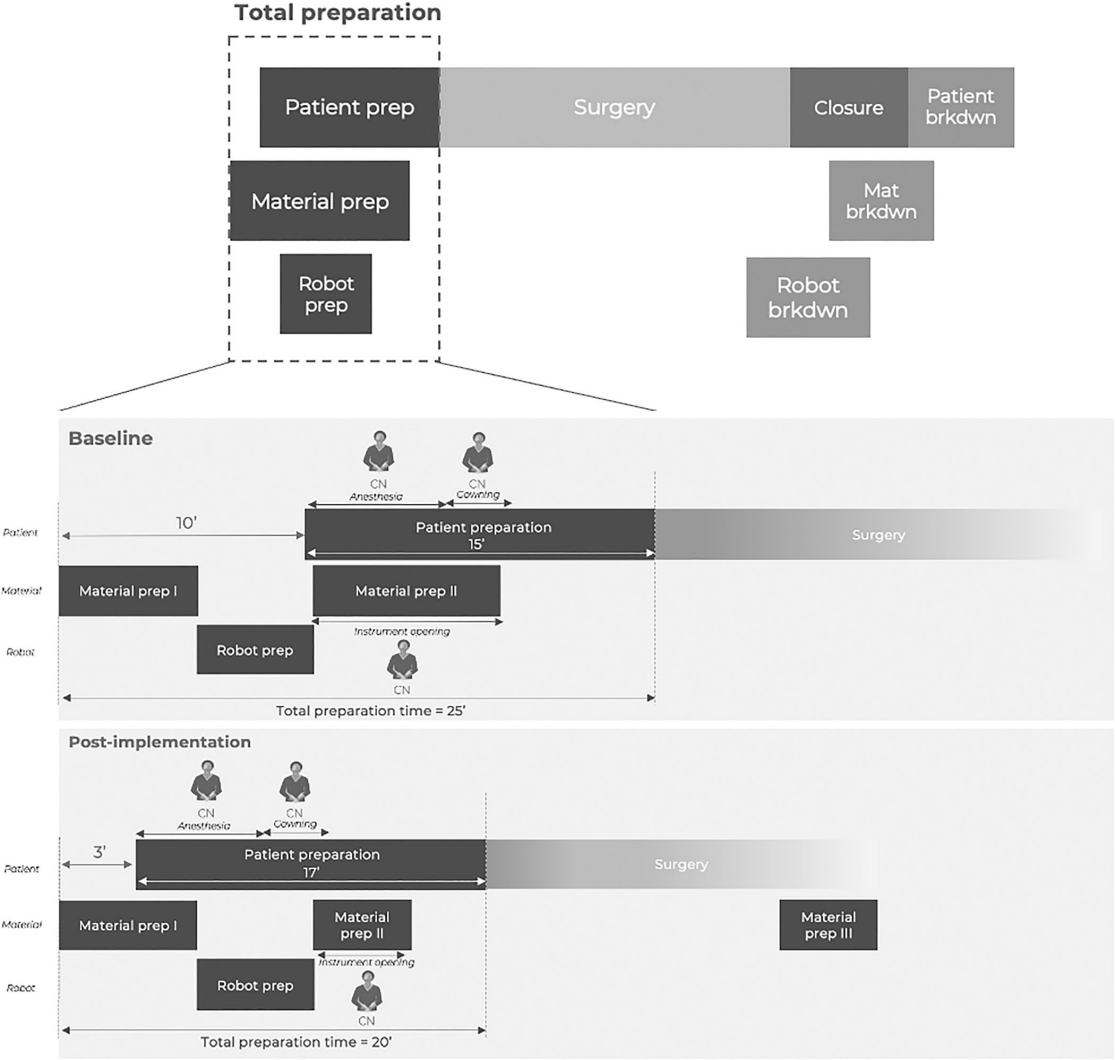
Detail of the total preparation time of baseline versus post‐implementation measurements. CN, circulating nurse.

After analyzing the baseline measurement data, it was observed that the circulating nurse had multiple responsibilities during the preparation phase. The nurse was responsible for several things including assisting the anaesthesiologist with anaesthesia, opening sterile sets to help the scrub nurse, and assisting the surgeon with sterile gowning (Figure 2). This increased responsibility not only caused idle times but also increased stress levels for the nurse. To address this issue, three procedural modifications were implemented.

First, the patient was brought into the OR seven minutes earlier on average, allowing for optimized parallelization of the patient, material and robot flow and securing the elimination of idle times. The simultaneous preparation of the robot (installation and draping) and the patient allowed for the circulating nurse to no longer perform concurrent tasks, but consecutive tasks, lowering the mental workload (Figure 2).

Second, the material preparation was split up. In the observed baseline measurements, material preparation consisted of two parts: material preparation I and material preparation II. During material preparation I, the consumables and the sets for robot preparation were opened. During material preparation II, all the other sets were opened. The procedural modification here was that only the sets required for the initial approach and knee registration with the robot were opened during material preparation II. The remaining sets, such as those that included the trial components, were opened during the approach and knee registration phases (material preparation III) (Figure 2).

**Figure 3:**
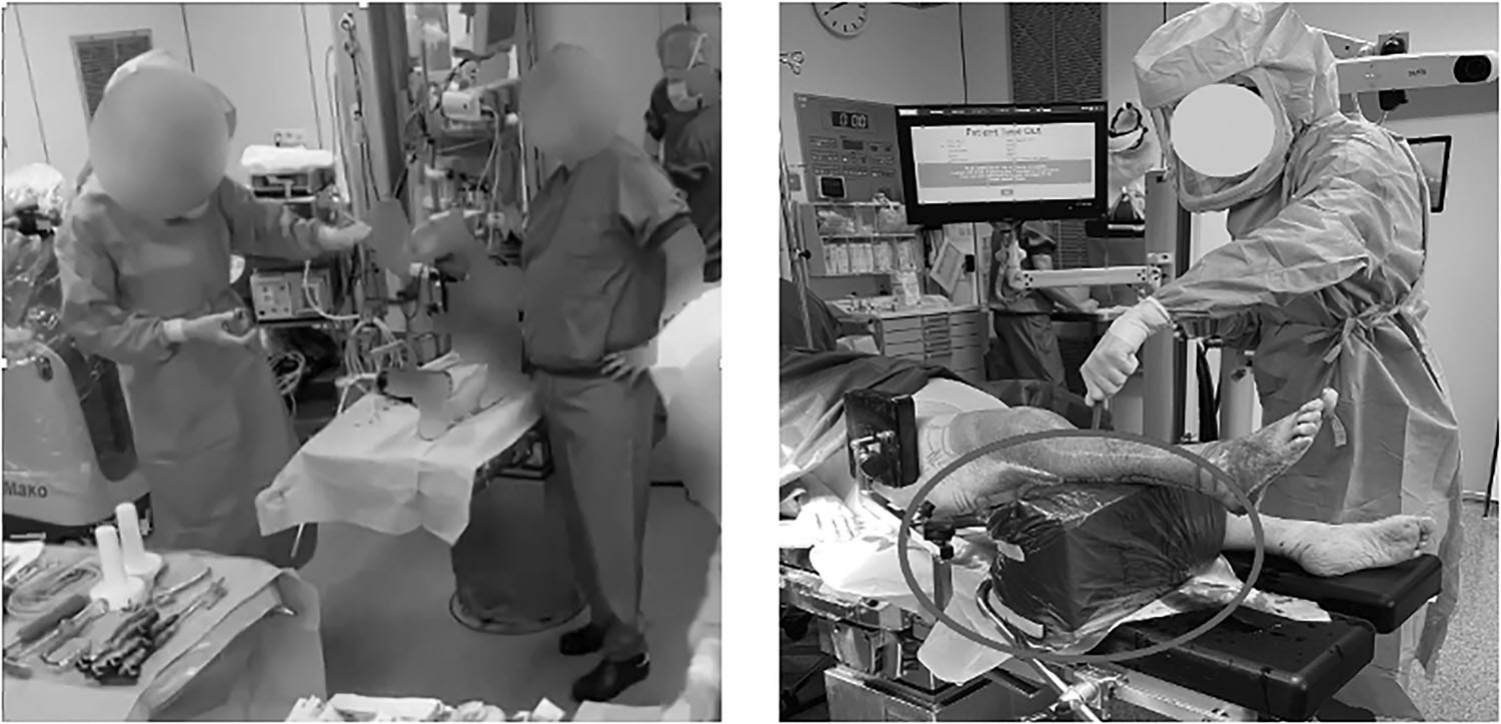
Left: The circulating nurse holding up the leg during disinfection during baseline measurements. Right: The use of a disinfection support device during disinfection during post‐implementation measurements.

Finally, a change was made during the disinfection of the patient's leg. Initially, the leg was held by the circulating nurse while the surgeon or performed disinfection (Figure 3). By replacing the nurse with a support device, this not only freed up time for the circulating nurse to assist with material preparation II to secure the parallelized flow but also reduced the physical workload for the circulating nurse.

Next to that, instrument analysis enabled the team to reduce the instrument trays. For procedures considered in the baseline measurement group, a total of eight (cemented TKA) or ten (cementless TKA) sets were opened during each procedure. The overall usage of the items and instruments in the sets was 29%, where 39% of all items were never used, and only 10% of all items were used during each procedure. Trial components were also included in this analysis.

**Figure 4:**
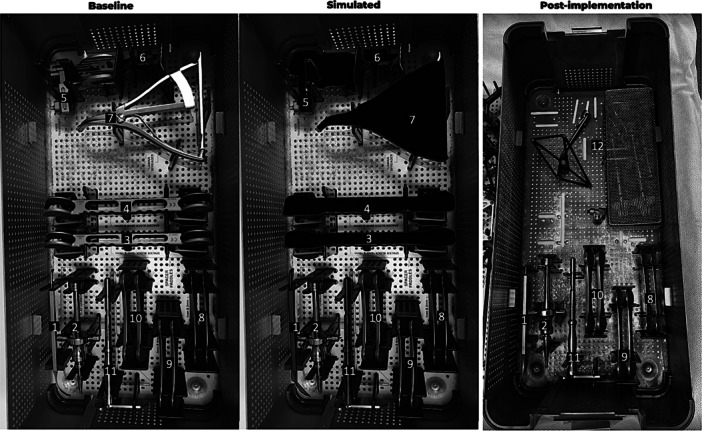
Example of eliminating instruments and rearranging trays.

**Figure 5:**
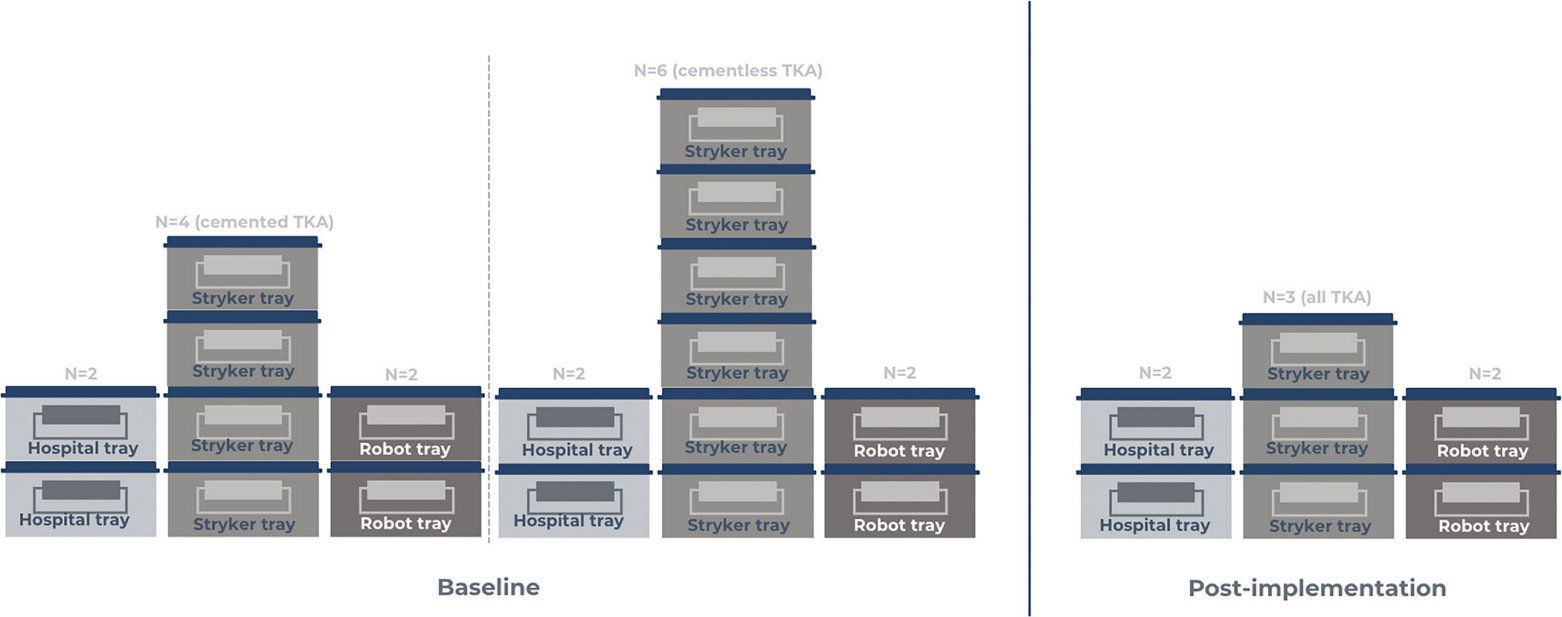
Overview of baseline (cemented and cementless TKA) and post‐implementation (all TKA) trays. TKA, total knee arthroplasty.

Figure 4 shows an example of the rearranging of one instrument tray. Instrument numbers 1 and 2 were used only 55% of the time, instrument numbers 3–7 were never used during procedures (0%), and instrument numbers 8–11 were always used (100%) (Figure 5—baseline). The simulation shows the elimination of instruments with 0% usage (Figure 4—simulated) making space in the tray to add other instruments (instrument number 12) (Figure 4—post‐implementation). After efficiency implementations for all sets, the number of sets opened per case was reduced to 7 sets (for both cemented and cementless TKA) (Figure 5). The reduction of instruments also leads to a mean weight reduction of 9.23 kg of material for each procedure.

### Time analysis

**Figure 6:**
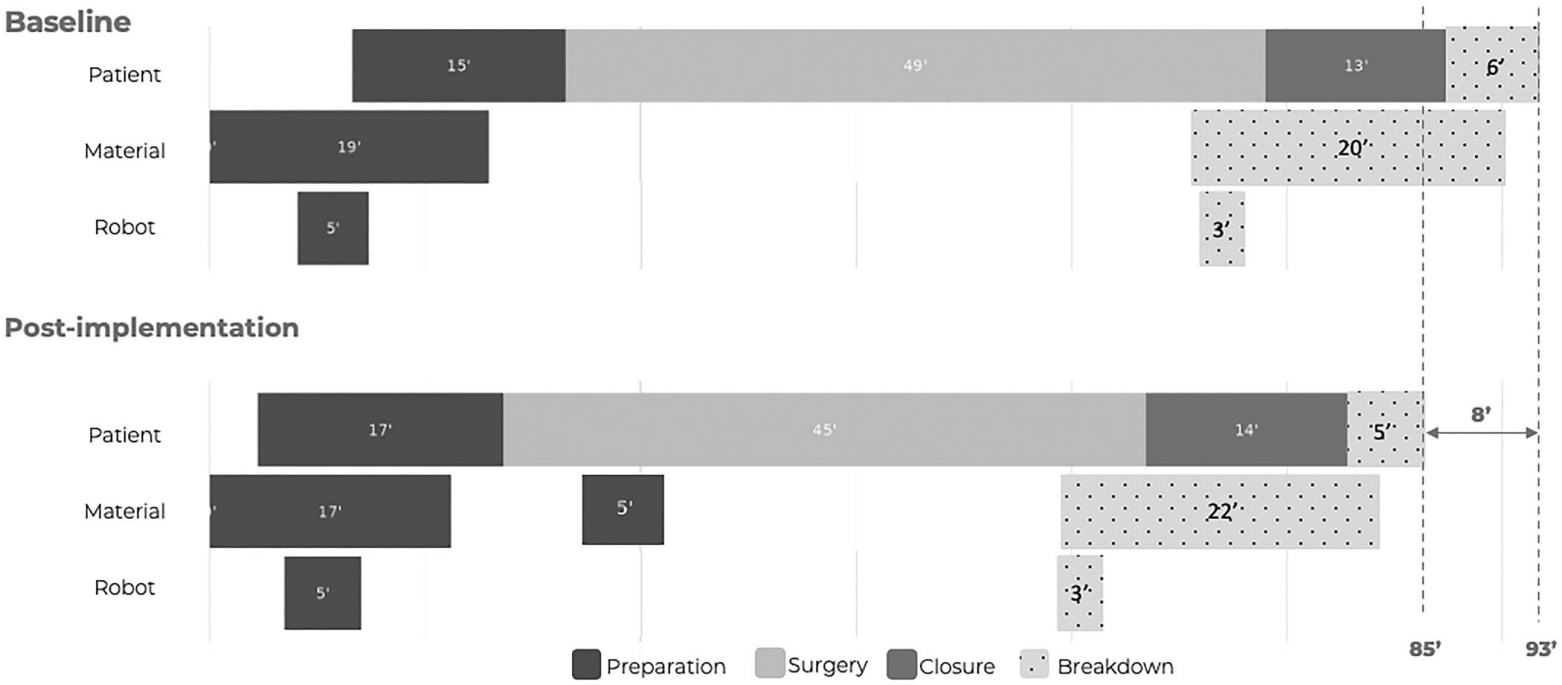
The comparison of total OR time between baseline measurements in 2023 (*n* = 25) and post‐implementation measurements in 2024 (*n* = 6).

Time analysis results are described in Table 2 and Figure 6. No significant differences were observed between both groups for total OR time (MD = 8.3 min, 95% CI = −0.7 to 17.4, *p* = n.s.), surgery time (MD = 3.2 min, 95% CI = −5.0 to 11.4, *p* = n.s.), breakdown time (MD = 1.0 min, 95% CI = −1.2 to 3.0, *p* = n.s.) and OR turnover (MD = 1.3, 95% CI = 1.3, 95% CI = −0.4 to 5.0, *p* = n.s.). There was a significant difference in preparation time between both groups (MD = 4.3, 95% CI = 1.3–7.3, *p* = 0.007), showing the efficiency gain during preparation after efficiency implementation. Although the total OR time and OR turnover time did not reach statistical significance (*p* = n.s.), there is an observable trend, as the 95% CI for the observed effect size narrowly includes 0. This suggests faster total OR time and a shorter OR turnover time in the post‐implementation group compared to the baseline. Further research with a larger sample size or additional controls may be warranted to explore this potential difference.

**Table 2:** Time analysis results.

	Baseline				Post‐implementation				Effect size		
OR timings	Mean ± SD	Range	Median	IQR	Mean ± SD	Range	Median	IQR	*p*	Mean difference	95% Confidence interval
Total OR time (min)	93.2 ± 10.1	71.9–113.1	94.5	87.0–98.4	84.8 ± 8.4	73.4–94.5	82.7	80.8–91.5	n.s.	8.3	−0.8 to 17.4
Preparation time (min)^a^	24.6 ± 3.3	17.6–32.0	25.7	22.6–26.2	20.3 ± 2.8	16.6–24.9	20.8	18.7–21.3	0.007^a^	4.3	1.3–7.3
Surgery time (min)	60.4 ± 9.2	78.8–42.2	60.7	55.0–66.1	57.2 ± 6.4	64.2–48.2	57.7	53.1–62.4	n.s.	3.2	−5.0 to 11.4
Breakdown time (min)	6.4 ± 2.4	12.1–2.1	5.9	5.0–7.1	5.5 ± 1.1	6.6–4.0	5.6	5.0–6.0	n.s.	1.0	−1.2 to 3.0
OR turnover (min)	9.9 ± 2.8	17.1–5.3	9.7	8.0–11.4	7.6 ± 1.5	9.8–5.9	7.5	6.7–8.3	n.s.	1.3	−0.4 to 5.0

Abbreviations: IQR, interquartile range; n.s., not significant; OR, operating room; SD, standard deviation.

^a^ Non‐parametric value based on Mann–Whitney *U* test.

## DISCUSSION

OR efficiency is an increasingly important topic in healthcare. Serhson et al. [21] and DeCook et al. [5] published about core efficiency principles to revolutionize OR management, as a model for enhancing OR efficiency. The application of lean methodologies maps out all OR processes, and let the OR teams reflect on those processes, performed in the OR for many years. Only then continuous improvement and efficiency gains are possible. Yet, the efficiency principles have not been validated in the literature with real‐world research data. In this study, we investigated whether applying the efficiency principles had an impact on OR efficiency.

A first efficiency principle is ‘time transparency’ [5], where effective allocation and utilization of time resources are crucial in the setting of the OR. By providing a clear view of how time is expended across various tasks and processes, an organization can uncover critical insights into operational dynamics. In this study, baseline measurement results showed the OR team a clear view of the time utilization of the entire OR process. This knowledge as a start helped identify bottlenecks and idle times, enabling the optimization of the process.

A second efficiency principle stated in literature is ‘the theory of constraints’ [5]. An OR is not run by a single person, the surgeon, but by an entire team of nurses, anaesthesiologists and fellow surgeons. Every single person in the team can be the bottleneck slowing down the process. The primary constraint is always the surgeon, ensuring that the surgeon's skills are utilized to their fullest potential without idle time and all processes and resources are aligned to support the surgeon's capacity, minimizing delays and inefficiencies. It is thought that having dedicated teams, specialized in specific procedures, is the key to reducing OR time and improving efficiency [8, 19]. However, this study shows that an important factor in improving OR efficiency is having a well‐documented procedure that is thoroughly understood by all team members. The specific responsibilities at every stage of the operation should be clear for every team member, which helps to minimize confusion and delays. If the procedure is well‐documented and consistently followed by the entire team, the perceived necessity of working with specific personnel becomes less critical. Even without the surgeon's preferred team, a structured and well‐coordinated procedure can deliver similar levels of efficiency.

A third efficiency principle is ‘parallel tasks’ [5]. Serial tasks often lead to inefficiencies; every sequential delay amplifies, extending wait times and reducing throughput. Implementing parallel tasks in the OR, such as patient, material and robot preparation simultaneously, can markedly improve operational flow. Effective parallel tasking minimizes idle time and maximizes the use of staff and resources. In this study, the baseline measurements showed that the team already did some parallel preparation, however an ineffective parallel tasking was identified, leading to multi‐responsibilities of the team. The action of shifting the patient preparation particular minutes earlier allows not only to parallelise the patient preparation and the robot preparation, but also allows the team to have consecutive tasks instead of concurrent tasks.

A fourth and last efficiency principle described in this article is ‘Work Breakdown Structure (WBS)’ [21]. WBS offers a proven blueprint for dissecting complex initiatives into more manageable tasks. Breaking down the entire OR process helps identify which small actions affect patient, material or robot flow and process efficiency. It allows staff to critically look at each part of their contribution to the flows and identify waste, redundancy, and ways to improve each individual step that is required. Resource allocation, particularly surgical instruments, is also an important focus of this efficiency principle. This study shows the reduction in weight and in the number of sterile instrument sets required per RATKA procedure, by removing unused items and rearranging sets, from 8–10 surgical instrument trays (baseline measurements) to 7 surgical instrument trays (post‐implementation measurements). This not only decreased the time required to open the sets but also lowered sterilization costs per procedure. Thus, streamlining instrument use not only enhances efficiency by simplifying workflows, but also lowers costs [4, 7, 16].

Several strengths of this study should be noted. First, all procedures were performed by the same surgeon, ensuring consistency in surgical technique. Additionally, different nurses were randomly allocated, which minimized any potential bias linked to staff familiarity with the procedure. The data collection process was also improved by using video recordings to capture the OR days, reducing the risk of measurement bias from real‐time observations made by an external person present in the OR.

Despite the strengths of this study, there are several limitations that must be considered. The most notable is the small and unequal sample sizes between the baseline and the post‐implementation groups, which may limit the generalizability of the results and affect the robustness of the statistical analysis. Additionally, while the study being conducted by a single surgeon ensures consistency in technique, this could also be viewed as a limitation, as findings from a single‐surgeon study may not be fully representative. The consistency in preparation, breakdown, and turnover times should ideally be reproducible across different surgeons within the centre.

## CONCLUSION

Introducing and optimizing OR efficiency has implications for reducing the total cost of care and improving patient experience, quality of care, and a healthy and sustainable work environment for the OR team. While manufacturing efficiency principles have been proposed to revolutionize OR management, these approaches lack validation through real‐world research. This study is the first to assess the impact of applying such principles, particularly in RATKA procedures, using systematic data collection through digital twin technology.

Findings indicate that better OR preparation, consistent staff allocation and effective staff training can reduce surgical times, minimize waste and improve OR throughput. Addressing primary constraints, parallelizing flows and breaking down processes decreases surgical wait times, enhances patient flow, and streamlines OR operations. Further research involving larger and more diverse cohorts is needed to validate these results and promote broader adoption of efficiency principles and practices in surgical settings.

## AUTHOR CONTRIBUTIONS

Conceptualization of the study, data analysis, manuscript drafting and manuscript revision: Akito Hiraoka. Data collection: Bart Swinnen. Manuscript drafting and manuscript revision: Aline Vandeputte. Data collection and statistical analysis: Willem Fransen. Conceptualization of the study, data collection, data analysis and manuscript revision: Geert Leirs.

## CONFLICT OF INTEREST STATEMENT

Geert Leirs is a consultant and reports personal fees from Stryker for educational purposes. The remaining authors declare no conflicts of interest.

## ETHICS STATEMENT

The Institutional Review Board for Medical Ethics (CME Noorderhart) approved the study in advance. All the patients provided written informed consent to use their data for research and publishing purposes.

## Data Availability

Upon reasonable request, the authors can provide access to the data used for all analyses.
